# Factors affecting pediatric isotonic fluid resuscitation efficiency: a randomized controlled trial evaluating the impact of syringe size

**DOI:** 10.1186/1471-227X-13-14

**Published:** 2013-07-24

**Authors:** Greg Harvey, Gary Foster, Asmaa Manan, Lehana Thabane, Melissa J Parker

**Affiliations:** 1 Department of Pediatrics, McMaster Children’s Hospital, McMaster University, 1200 Main St W. Room 3A, Hamilton, ON, Canada; 2 Department of Clinical Epidemiology and Biostatistics, McMaster University, 1200 Main St W., Hamilton, ON L8N 3Z5, Canada; 3 Biostatistics Unit,/FSORC, St Joseph’s Healthcare Hamilton, 3rd floor Martha Wing, 50 Charlton Avenue East, Hamilton L8N 4A6, Canada; 4 Department of Anesthesia, McMaster University, 1200 Main St W., Hamilton, ON L8N 3Z5, Canada; 5 Division of Emergency Medicine, Department of Pediatrics, the Hospital for Sick Children, University of Toronto, 555 University Avenue, Toronto, ON M5G 1X8, Canada

**Keywords:** Fluid therapy, Resuscitation, Shock, Pediatrics

## Abstract

**Background:**

Goal-directed therapy guidelines for pediatric septic shock resuscitation recommend fluid delivery at speeds in excess of that possible through use of regular fluid infusion pumps. In our experience, syringes are commonly used by health care providers (HCPs) to achieve rapid fluid resuscitation in a pediatric fluid resuscitation scenario. At present, it is unclear which syringe size health care providers should use when performing fluid resuscitation to achieve maximal fluid resuscitation efficiency. The objective of this study was therefore to determine if an optimal syringe size exists for conducting manual pediatric fluid resuscitation.

**Methods:**

This 48-participant parallel group randomized controlled trial included 4 study arms (10, 20, 30, 60 mL syringe size groups). Eligible participants were HCPs from McMaster Children’s Hospital, Hamilton, Canada blinded to the purpose of the trial. Consenting participants were randomized using a third party technique. Following a standardization procedure, participants administered 900 mL (60 mL/kg) of isotonic saline to a simulated 15 kg child using prefilled provided syringes of the allocated size in rapid sequence. Primary outcome was total time to administer the 900 mL and this data was collected through video review by two blinded outcome assessors. Sample size was predetermined based upon a primary outcome analysis using one-way ANOVA.

**Results:**

12 participants were randomized to each group (n=48) and all completed trial protocol to analysis. Analysis was conducted according to intention to treat principles. A significant difference in fluid resuscitation time (in seconds) was found between syringe size group means: 10 mL, 563s [95% CI 521; 606]; 20 mL, 506s [95% CI 64; 548]; 30 mL, 454s [95% CI 412; 596]; 60 mL, 455s [95% CI 413; 497] (p<0.001).

**Conclusions:**

The syringe size used when performing manual pediatric fluid resuscitation has a significant impact on fluid resuscitation speed, in a setting where fluid filled syringes are continuously available. Greatest efficiency was achieved with 30 or 60 mL syringes.

**Trial registration:**

ClinicalTrials.gov, NCT01494116

## Background

Pediatric shock is a recognized medical emergency [1]. Aggressive fluid resuscitation is recognized as a critical component of early non-cardiogenic shock management [1-4]. The American College of Critical Care Medicine (ACCM) guidelines for early goal-directed pediatric septic shock management recommend to, “Push boluses of 20 cc/kg isotonic saline or colloid up to & over 60 cc/kg until perfusion improves or unless rales or hepatomegaly develop” [4]. Clear pragmatic recommendations for health care providers (HCPs) as to how to achieve rapid fluid resuscitation are lacking in current guidelines. An important aspect limiting fluid flow is that the intravenous (IV) cannulas most commonly used in pediatric patients have a small radius relative to those used in adults [5].

In adult patients, options for rapid fluid resuscitation include rapid infuser devices and pressure bag support [6-8]. While these modalities are available for use in pediatric resuscitation, in our experience, syringes are most commonly used for this purpose, likely due to their relative availability and health care providers’ comfort using them. A randomized controlled trial by Stoner et al. determined that manual syringe and pressure bag methods were an equivalent means of delivering fluid rapidly in an emergency department setting [9]. However, in this study only 58% of children resuscitated in the pressure bag and 68% in the syringe group met the ACCM benchmarks.

In the clinical setting, we have observed and health care providers have endorsed two different manual syringe techniques used for the purpose of rapid isotonic fluid resuscitation for children in shock: 1) the ‘disconnect-reconnect’ technique and 2) the ‘push-pull’ technique [10]. When initiating manual fluid resuscitation using syringes, a health care provider must make a decision regarding what syringe size to use. A larger syringe has a larger radius relative to a given IV catheter. To create the same pressure gradient (which is proportional to flow rate) across an IV catheter, a health care provider must apply a comparatively greater force to the plunger when a larger syringe size is used, as dictated by the formula. F = ΔP(πr^2^). Where the force applied is constant, a slower fluid flow rate results when a larger radius syringe is used.

**Figure 1:**
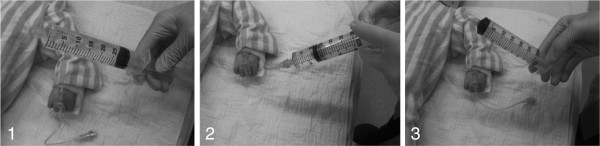
**The ‘disconnect-reconnect’ technique for rapid fluid resuscitation.** This method involves **(1)** connecting a fluid filled syringe to the IV extension tubing, **(2)** administering the fluid manually, and then **(3)** disconnecting the empty syringe, thus allowing for connection of another ready syringe that has been filled with fluid by an assistant, and repeating until fluid delivery is completed.

When using the ‘disconnect-reconnect’ technique (Figure 1) to perform manual fluid resuscitation, one must also consider that total fluid administration time is actually the sum of the “fluid push time” plus the “syringe swap time”, as time is required to disconnect and replace empty syringes with new fluid filled ones. Based on these considerations, while the actual “fluid push time” may be slower with use of larger syringes, it remains unclear which syringe size is most efficient in terms of total fluid administration time because the smaller syringes need to be exchanged more frequently, proportionally increasing the “syringe swap time”. Although the ‘disconnect-reconnect’ technique is crude, it is commonly practiced in our experience and interestingly not previously reported in the literature. The objective of our study was therefore to determine whether an optimal syringe size exists to facilitate rapid pediatric fluid resuscitation using the ‘disconnect-reconnect’ technique of manual fluid administration.

## Methods

The study was a single-blind, non-clinical, parallel group randomized controlled trial with four study arms. The trial was conducted at McMaster Children’s Hospital, a tertiary pediatric academic center in Hamilton, Canada. Approval for study conduct was obtained from the Faculty of Health Sciences/Hamilton Health Sciences Research Ethics Board. Written informed consent was obtained from all participants prior to participation. Although a non-clinical trial, we elected to register this study at www.ClinicalTrials.gov (NCT01494116). Conduct of this trial was supported by funds obtained from the Department of Pediatrics.

### Study participants

Eligible participants included staff physicians, postgraduate trainees, and nurses who were recruited by an e-mail and poster campaign. We excluded non-English speaking individuals and those incapable of performing manual fluid administration with a syringe. Gift certificates ($25 coffee card) were offered to each subject as a participation incentive. To further motivate peak performance among subjects, further prizes were awarded for those with the fastest fluid administration times. Participants were only allowed to participate on one occasion.

### Randomization, allocation and blinding

Participants were assigned to one of four study arms, in 1:1:1:1 ratio, using a third party randomization technique. The independent third party created the randomization schedule using http://www.randomization.com and kept this secret from and inaccessible to the investigators. Allocation was therefore concealed. The randomization schedule utilized permuted blocks of randomly varying size.

Participants were provided with details regarding the trial sufficient to achieve informed consent however they were not advised of the hypotheses of the investigators. It was not possible to blind the research assistants, as they needed to be familiar with the study protocol and administer the intervention. Primary outcome assessment was blinded in that the two individuals responsible for extracting outcome data from the trial video recordings were not familiar with trial details or its purpose. The outcome assessors were not advised of subject group assignment and only had access to the study ID number associated with a given video recording. They were also unaware of other data collected as part of the trial. Collected data was input by GH, with verification by MP. The research assistants conducting the trial were conceivably aware of the purpose of the trial (though had no conflicts of interest with respect to the study outcomes). All investigators had access to the raw data. We therefore considered our trial as single-blind.

### The model

The model in this study is best described as a low fidelity simulator rendered “physiologic” in that it incorporated a 1.00-inch, 22-gauge IV catheter. Catheters of this size are used clinically in newborn to adolescent age patients, making this a rational choice for our purposes. Please see Additional file 1 for figures of the model and a detailed description.

### Intervention procedure

Following written consent, participants were randomized to one of the four syringe size groups (10, 20, 30, or 60 mL). Upon receiving the participant group assignment, a research assistant measured 900 mL of 0.9% normal saline using a graduated cylinder and then this was divided into three 300 mL aliquots (each aliquot 20 mL/kg based on a 15 kg simulated patient). Each aliquot was then drawn up into colour-coded syringes of the assigned size, with each of the three aliquots having a unique assigned colour. Syringes of the same colour were then placed in each of three kidney bowls.

To ensure adequate familiarity with study procedures and the model prior to formal testing, all subjects underwent a standardization procedure [11]. This consisted of a brief video orienting participants to the study setting and demonstrating the ‘disconnect-reconnect’ technique. Subjects were then provided with 3 fluid-filled demonstration syringes and given a brief opportunity to practice the technique. Following this, subjects were verbally presented with a clinical vignette of a febrile 15 kg toddler in decompensated septic shock in need of immediate rapid fluid resuscitation. They were advised to administer the fluid using the provided syringes as rapidly as possible, finishing each 20 mL/kg colour set in sequence. Trials were commenced on verbal prompt by the research assistant and proceeded without interruption.

All subject testing was video recorded in a manner which captured the manual performance of fluid administration for outcome ascertainment purposes, but which did not capture participant identifiers. In addition to video recording all testing, the research assistant timed with a stopwatch the initial two participant trials. Due to clear inaccuracies with use of the stopwatch timing method, we reverted to use of the trial video recordings for outcome ascertainment as per our a priori plan. A data collection form was completed by the research assistant at the time of testing to record other secondary outcomes of interest. Each participant also completed a post-trial questionnaire.

### Outcome measures and assessment

Fluid administration times (in seconds) were determined from video review by two independent outcome assessors blinded to the purpose of the trial. For each video, the assessors were asked to determine four separate fluid administration times based upon a clear, a priori defined protocol to ensure consistency. A common software program (Apple Quicktime™) was used to review the trial videos and the time bar function was used to identify times in the video frame sequence. Time outcomes extracted included time to administer the full 900 mL (60 mL/kg) of NS (primary outcome measure) and times to administer each of the three 300 mL (20 mL/kg) aliquots of NS, (secondary outcome measure). For the purposes of final data analysis, the times of the two independent assessors were averaged for each outcome of interest.

Descriptive data regarding the characteristics of participants were ascertained from the post-trial questionnaire. The questionnaire also asked participants to recall and rate their level of fatigue following each 20 mL/kg bolus on a 7-point Likert scale. Catheter dislodgement events (defined as physical displacement/removal from the conduit tubing) were noted by the research assistant during testing and on the data collection form. The volume of normal saline actually received by the model was determined by the research assistant by measuring the amount of fluid collected in the graduated cylinder.

### Statistical analyses and sample size considerations

The analysis results of subject baseline characteristics and outcome variables (both primary and secondary) were summarized using descriptive summary measures: expressed as mean (standard deviation) or median (minimum-maximum) for continuous variables and number (percent) for categorical variables. Final statistical analyses were performed using SAS (SAS Institute Inc., Cary, NC, USA), although SPSS (IBM Corporation, Armonk, NY, USA) was used for some preliminary analyses and figure generation.

The primary outcome was analysed using a One-way ANOVA analysis, with post hoc comparison of syringe group total intervention time means using Tukey’s HSD. Secondary outcome analyses consisted of Generalized Linear Model (GLM) with repeated measures in order to compare bolus administration times and fatigue scores for each of the three sequential aliquots. We planned to use Chi-square testing to compare the proportion of catheter dislodgement events by syringe size group. One-way ANOVA was used to compare the mean volume of normal saline received by the model according to syringe size group. A two-way random effects model was used to compare the agreement of our blinded outcome assessors (both observer and subject were treated as random effects) [12].

The 48 subject sample size for the trial was determined a priori based upon the planned One-way ANOVA primary outcome analysis. Sample size calculations utilized an estimated effect size, determined based upon preliminary testing in the model. Using a significance level of 0.05 and power of 80%, the sample size needed for the trial was conservatively estimated at 12 subjects in each group, 48 total.

## Results

**Figure 2:**
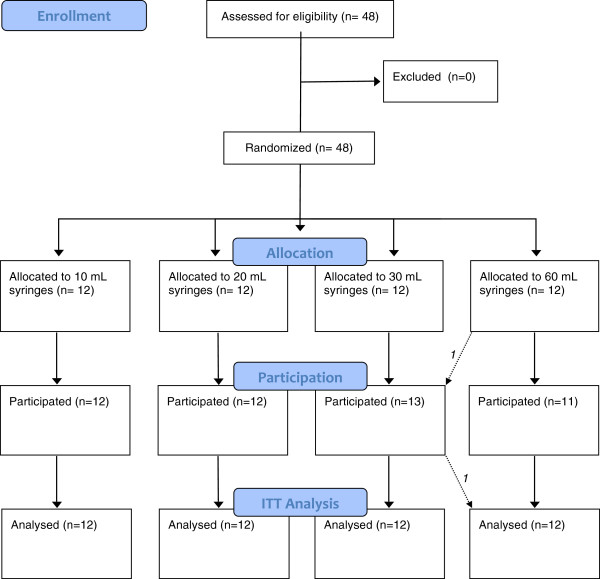
**The Pediatric Fast Fluid Trial flow diagram.** No participants were excluded from initial 48 subject recruitment. All subjects completed protocol to analysis. Initial allocation called for 1:1:1:1 syringe size distribution, however one subject was mistakenly allocated to participate in the 30 mL group from the 60 mL group. This same subject was analysed as per protocol in the 60 mL group after per protocol analysis showed no change in primary outcome.

Forty-eight participants were recruited from October 2011 to December 2011 with no excluded participants. The process of subject selection and flow throughout the study is summarized in a flow-diagram in accordance with the CONSORT Statement (Figure 2) [13,14]. Notably, one participant who should have been assigned to the 60 mL group according to the randomization sequence was incorrectly allocated to and received the 30 mL assignment due to a communication error. This individual was analyzed in the 60 mL group as per intention to treat principles but we also conducted a per protocol analysis of the primary outcome to assess for any potential impact this may have had on the primary outcome. The per-protocol analysis failed to show any difference in the primary outcome analysis result. Further study analyses were therefore conducted using only an intention-to-treat analysis.

**Figure 3:**
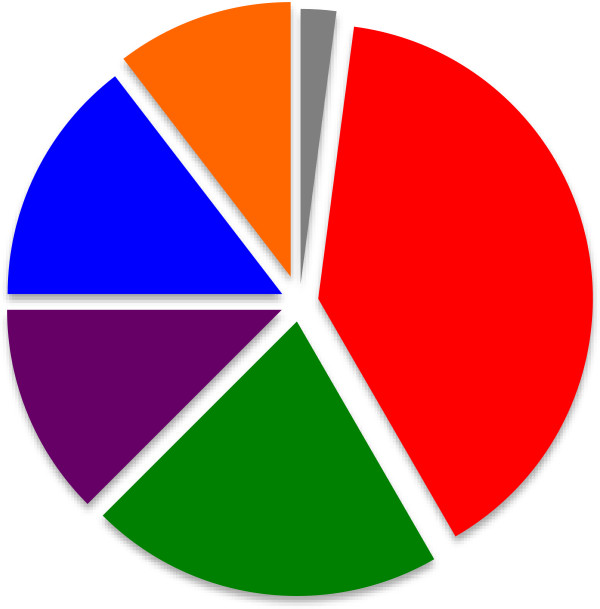
**Preferred techniques of rapid fluid resuscitation as reported by participants.** The majority of respondents reported preference for the ‘disconnect-reconnect’ technique of fluid bolusing. The next most commonly cited preference was the ‘push-pull’ technique, and 8/48 participants reported preference for a regular infusion pump – an unacceptable means of delivering a fluid bolus in a resuscitation scenario. Legend: gray, Pressure bag; red, Disconnect-Reconnect technique; green, Push-Pull technique; violet, Regular infusion pump; blue, Don’t know/no answer; orange, More than one preferred technique.

Baseline demographics of the participants as gathered from the post-intervention questionnaire are seen in Table 1. Participants indicated that they were most comfortable using the ‘disconnect-reconnect’ technique as their preferred method of fluid administration for children in shock (48%), though many also preferred the ‘push-pull’ technique (27%); regular infusion pump was also preferred by 14% (Figure 3). Respondents were asked to choose one preferred method, however several circled more than one answer on their post-test questionnaire (48 subjects provided 54 responses). Four respondents did not provide an answer.

**Table 1:** Baseline demographics of trial participants

**Variable**	**Syringe group**				**Total**
	**10**	**20**	**30**	**60**	
Age Range					
>50	1	1	0	0	2
40-49	1	2	4	2	9
30-39	2	6	5	6	19
20-29	8	3	3	4	18
Profession					
Nurse	5	9	5	7	26
Nursing Student	1	0	0	0	1
Resident	4	1	4	4	13
Staff Physician	2	2	3	1	8
Fluid Resuscitation Experience					
None	2	0	2	0	4
Minimal	3	0	1	2	6
Some	2	2	2	3	9
Experienced	1	6	2	3	12
Very Experienced	4	4	5	4	17
*Use Syringes for Fluid Resuscitation					
Yes	10	9	8	11	38
No	0	3	1	1	5
Don’t Know	0	0	1	0	1
*Use ‘disconnect-reconnect’ Technique					
Yes	7	9	8	11	35
No	3	3	0	0	6
Don’t Know	0	0	2	1	3

*Answers were not asked of four participants whom wrote ‘none’ to fluid resuscitation experience.

**Figure 4:**
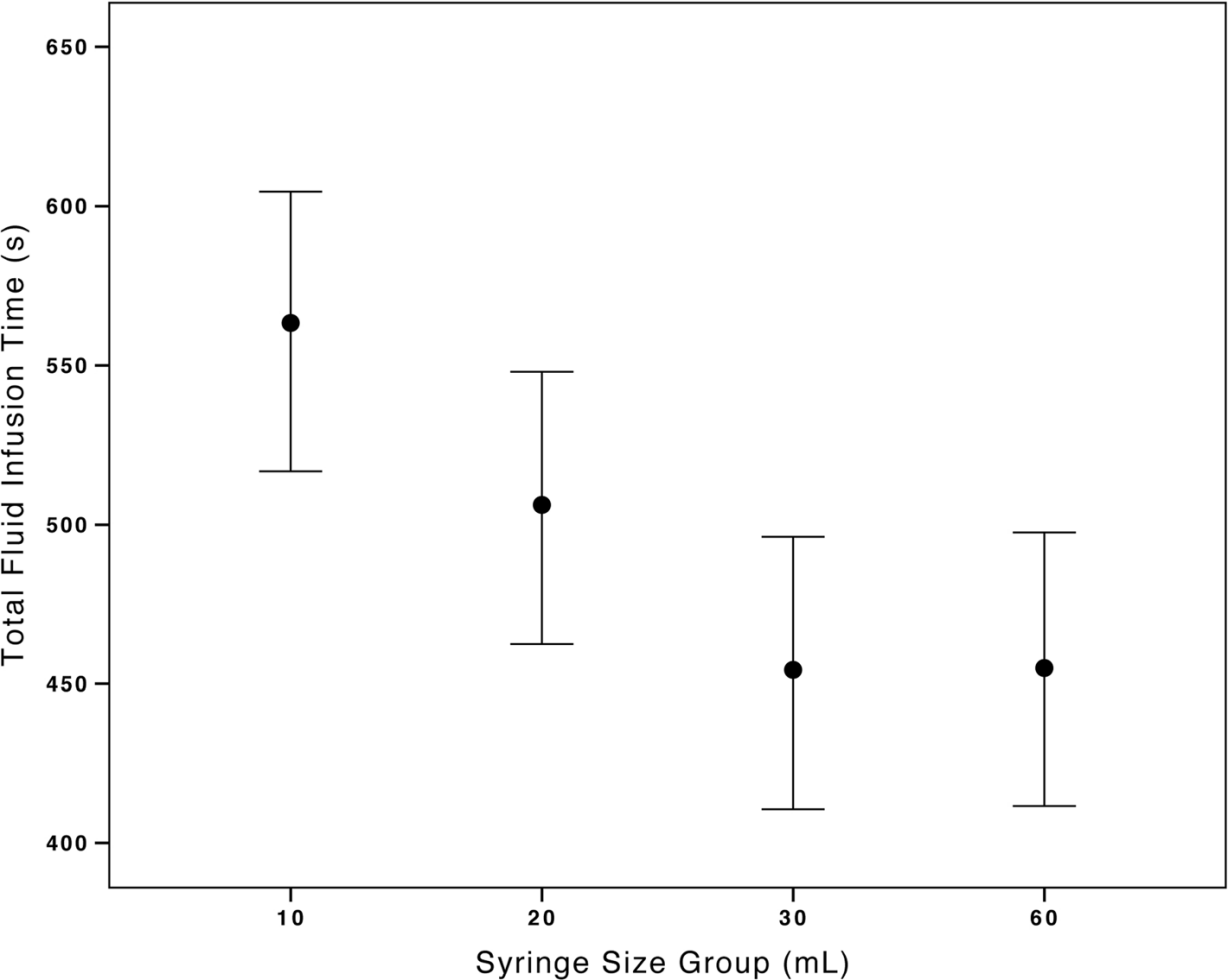
**Mean fluid bolus times by syringe size with 95% confidence intervals (primary outcome analysis).** Significant difference between 10 mL and 30/60 mL syringe size groups is clearly demonstrated. There is a notable trend of superiority between of the 30/60 mL groups over the 20 mL group, but this did not achieve significance. This trend provides empirical validity to PALS recommendations suggesting use of 35–60 mL syringes for optimal fluid resuscitation [1].

The primary outcome of total fluid delivery time significantly differed according to syringe size based on our analysis with one-way ANOVA at p = 0.0012 (Table 2). Post Hoc analysis with Tukey’s HSD demonstrated a significant difference in fluid administration time when comparing the 10 mL group to both the 30 mL and 60 mL groups respectively (Table 3). There did appear to be a trend towards superiority of the 30 mL and 60 mL groups over the 20 mL group, but this was not statistically significant (Figure 4).

**Table 2:** **Total mean fluid bolus times and mean rates with 95**% **confidence intervals by syringe size grouping**

**Syringe group**	**N**	**Mean time (s)**	**95% CI**	**Mean rate (mL/s)**	**95% CI**
10	12	563.50	[521.43, 605.57]	1.62	[1.47, 1.78]
20	12	506.42	[464.34, 548.49]	1.81	[1.66, 1.97]
30	12	454.42	[412.34, 496.49]	2.04	[1.89, 2.19]
60	12	454.92	[412.84, 496.99]	1.99	[1.84, 2.14]

One-way ANOVA between groups significant at p = 0.0012.

Mean rates reported for comparison sake to other literature.

**Table 3:** Tukey HSD comparison testing of syringe group mean total bolus times

**Syringe group:**	**Comparison group:**	**Significance (p-value)**
10	20	0.228
	30*	0.003
	60*	0.003
20	10	0.228
	30	0.306
	60	0.315
30	10*	0.003
	20	0.306
	60	1.000
60	10*	0.003
	20	0.315
	30	1.000

* - The mean difference is significant at the 0.05 level by Tukey HSD test comparison.

**Figure 5:**
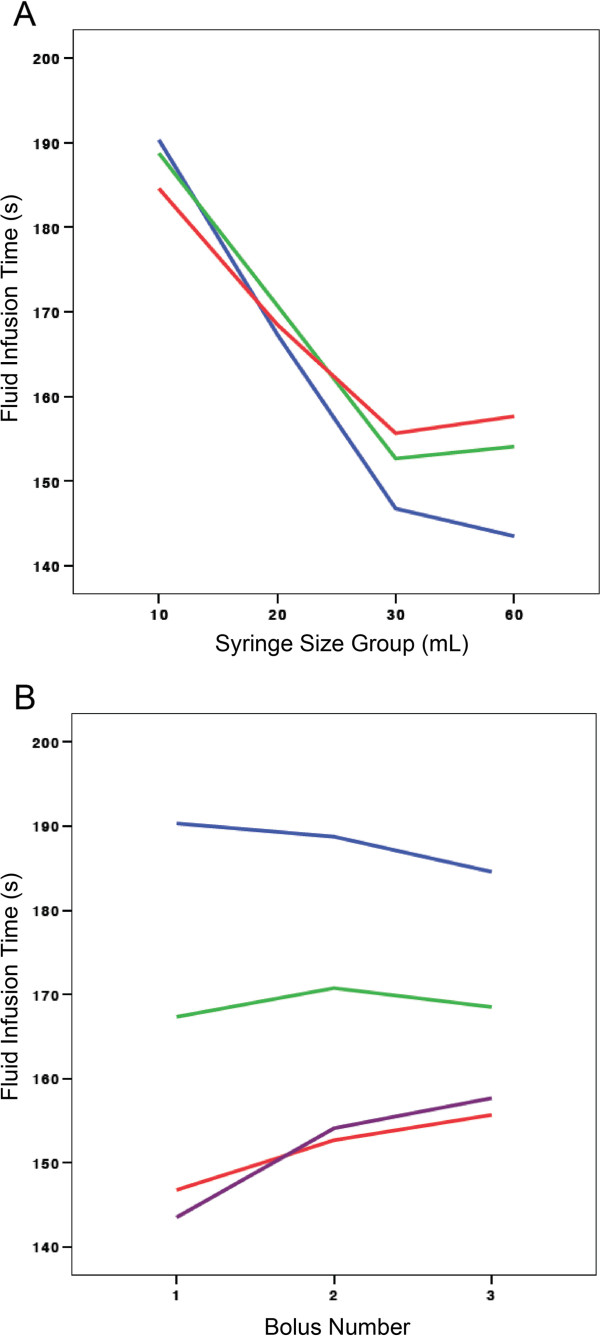
**A Fluid infusion time by syringe size group.** In the GLM analysis an interaction was found between syringe size group and bolus number that precluded comment on the impact of bolus number on fluid infusion time. This outcome was intended to determine whether progressive fatigue objectively occurred among providers with repeated fluid bolus administration. **A**: blue, First 20 mL/kg normal saline bolus; green, Second 20 mL/kg normal saline bolus; red, Third 20 mL/kg normal saline bolus. **B** Fluid infusion time by bolus number. This figure assists with understanding why the interaction between syringe size and bolus number is occurring. It appears that the 10 mL syringe size group “speeds up” in terms of the time to administer sequential 20 mL/kg fluid boluses while the larger syringe size groups appear to “slow down”. The 10 mL group likely “sped up” because providers became more efficient at rapidly disconnecting and reconnecting syringes. **B**: blue, 10 mL syringe group; green, 20 mL syringe group; red, 30 mL syringe group; violet, 60 mL syringe group.

**Figure 6:**
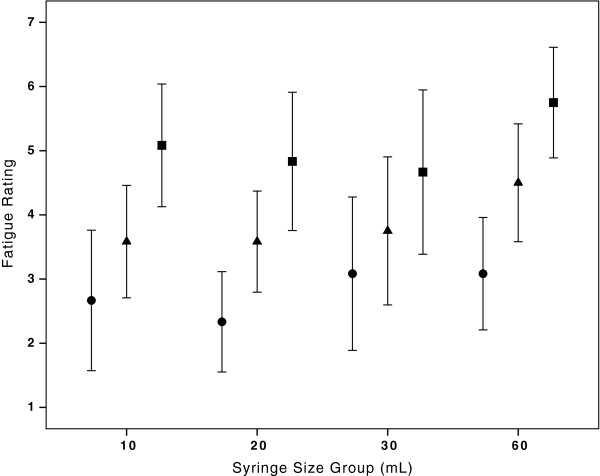
**Mean fatigue score with 95% confidence interval by syringe size group and bolus number.** Increased fatigue scores correlated significantly with bolus number in each syringe group by GLM analysis. This provides a subjective basis for our recommendation to consider provider changes during a fluid resuscitation. Legend: ●First 20 mL/kg normal saline bolus; ▲Second 20 mL/kg normal saline bolus; ■Third 20 mL/kg normal saline bolus.

The GLM analysis to assess bolus time by bolus number detected an interaction between syringe size and bolus number (Figure 5). As a consequence, we are unable to report the main effect related to this outcome of interest. The GLM analysis, with Greenhouse-Geisser correction, for HCP self-reported fatigue by bolus number did differ significantly across bolus 1, 2, and 3 (F 120.19, p<0.0001). There was no significant interaction in this analysis (Figure 6). Syringe size did not have a statistically significant impact on fatigue scores (p=0.51).

The total amount of fluid received by the model as a result of resuscitation was not significantly different between syringe size groups (p=.177) (Table 4). There were no catheter dislodgement events and so this outcome was not analyzed. Excellent agreement was found between the two blinded outcome assessors based on the total fluid administration time data extracted from the trial video recordings (ICC=0.99997).

**Table 4:** **Total mean cylinder volumes with 95**% **confidence intervals by syringe group**

**Syringe group**	**N**	**Mean volume: (mL)**	**95% CI**
10	12	872.92	[866.795, 879.039]
20	12	872.22	[866.211, 878.455]
30	12	870.75	[864.628, 876.872]
60	12	864.25	[858.128, 870.372]

One-way ANOVA between groups not significant at p = 0.1771.

## Discussion

This trial demonstrates a significant impact of syringe size on fluid administration time in a study setting involving health care provider subjects and a non-clinical pediatric fluid resuscitation model. Our results suggest that the use of larger syringe sizes (30 mL or 60 mL) is most efficient and dissuades the use of 10 mL syringes in situations where rapid pediatric fluid resuscitation is required. While the 20 mL syringe size was not statistically inferior to the 30 and 60 mL sizes, there was a trend towards inferiority and the 20 mL group results did not statistically differ from the 10 mL group.

We had hypothesized that HCPs would objectively fatigue over the course of performing the intervention as borne out by differences in the administration times of boluses 1, 2, and 3. We were unable to confirm or refute this hypothesis due to the presence of an interaction that precluded assessment of the main effects in this analysis. There was, however, a statistically significant increase in HCP self-reported fatigue with successive fluid boluses. Examining our results graphically by syringe group, it appears as if there is a trend toward increased self-reported fatigue among providers using the 60 mL syringe size (Figure 6). This makes sense with what we know about the physics and physiology: it is physically more difficult for providers to depress the syringe plunger of fluid filled syringes of a larger diameter.

While the presence of an interaction prevented us from assessing an impact on fluid administration time by bolus number, the interaction graph is itself interesting and somewhat instructive (Figure 5). The interaction appears to have occurred because the 10 mL group seemed to “speed up” with time, while the other 3 syringe size groups appear to have slowed down with ongoing fluid resuscitation. One hypothesis that may be generated from this finding is that individuals in the 10 mL group may have become more efficient at connecting and disconnecting the syringes over the course of the intervention. Because the 10 mL group had the greatest number of syringes to connect and disconnect for each bolus, proportionally speaking, the time allocated to disconnecting and reconnecting syringes was greatest for this group. In contrast, the observation that the other syringe size groups appeared to slow down with time would fit with our a priori hypothesis of provider fatigability.

Our finding of progressive subjective fatigue among trial participants is certainly noteworthy and not previously reported in the literature. In other physically strenuous resuscitative tasks, such as the performance of chest compressions (CPR), current best practices involve frequent provider switches to avoid performance decay [15]. We suggest that given how fatiguing rapid manual fluid administration can be, perhaps routine provider switches are warranted for this resuscitation task as well. This issue is not addressed in current resuscitation guidelines. A logical time for provider switches would be between 20 mL/kg boluses.

The finding that a number of our study participants believed that regular infusion pumps were an adequate pediatric fluid resuscitation method underscores that more education is needed for HCPs regarding optimal fluid resuscitation performance. We still encounter standard IV pumps being inappropriately utilized in the setting of shock. Such pumps provide a maximum fluid delivery rate of 999 mL/hr, which in almost all cases is insufficient to achieve ACCM benchmarks. For example, for a 15 kg child, as simulated by our model, a 20 mL/kg bolus would take 18 minutes to infuse with use of a regular IV pump. As such pumps are often the most convenient means to operationalize a fluid bolus order, it is imperative for the physicians writing such orders to be explicit regarding the intended time frame and method of administration.

There are several limitations to our trial that warrant mention. Firstly, in a real resuscitation, syringes are not neatly prepared as was the case in our trial. However, with use of the ‘disconnect-reconnect’ method, syringes are typically prepared quickly by one or more HCPs resulting in a similarly “limitless” supply - that often accumulates. Second, while use of a crossover design may have been preferable, with 4 intervention groups, we felt that use of this design would negatively impact on feasibility and increase the risk of participant dropout. We are satisfied that participants’ characteristics appear well balanced across the groups in our study. Thirdly, no catheter dislodgement events were recorded in our trial. It is possible that features attributable to our model had a protective effect against catheter dislodgement, although this was indeed possible and occurred during pilot testing. In this context, it is notable that only 1/57 subjects in the clinical trial performed by Stoner et al. experienced a catheter failure [9]. Finally, our trial protocol did not strictly adhere to ACCM guideline insofar as “patient” reassessments between each 20 mL/kg bolus are recommended [4]. In our experience, these reassessments often do not slow HCPs from administering fluid where ongoing resuscitation in required and such assessments are often done concurrently.

Although our study was conducted in the non-clinical setting, we had typical health care providers perform rapid fluid administration as they would under resuscitative conditions. The model incorporated an IV catheter and so resistance to fluid flow was as it would be in the clinical setting. Further, infants and children with decompensated shock, as in our clinical vignette are typically lethargic and so patient movement may not be all that dissimilar to our model. We therefore believe that our findings can likely be cautiously extrapolated to the clinical setting.

Our conclusions, and any other optimizations to be made in rapid fluid resuscitation relate to statistically significant differences in the order of seconds to minutes. Therefore, ultimately demonstrating whether improvements in pediatric fluid resuscitation performance have an impact on patient important outcomes like morbidity and mortality may be difficult. Nonetheless, observational studies have provided the basis for current goal-directed ACCM benchmarks, [16,17] and subsequent prospective studies have shown morbidity and mortality benefit with adherence to these [18,19].

Morbidity and mortality related to pediatric septic shock has dropped significantly in recent decades – owing in part to improved recognition and aggressive management, of which fluid resuscitation is currently considered a critical component [20,21]. While studies such as the FEAST trial [22] have begun to raise questions regarding the role and extent of fluid resuscitation in the treatment of septic shock, the purpose of our study was not to challenge current ACCM guidelines, for which support has recently been reaffirmed [23]. Instead, we sought to evaluate and improve upon implementation, which is known to be problematic [17-19]. Furthermore, the need to increase clarity and pragmatic instruction for health care providers regarding how best to perform fluid resuscitation is relevant to the management of all forms of non-cardiogenic shock.

## Conclusions

Our results support use of 30 and 60 mL syringes for the purposes of rapid pediatric fluid resuscitation when the ‘disconnect-reconnect’ technique is utilized. Further studies are needed to evaluate the comparative efficiency of other fluid resuscitation techniques, the potential problem of provider fatigability, and how fluid resuscitation is best performed in the context of multi-provider teams. An improved body of evidence should assist with generating clear best practice recommendations as to how pediatric fluid resuscitation is best performed.

### Key messages

1) When using a syringe for pediatric fluid resuscitation, choose a 30 – 60 mL syringe.

2) Regular infusion pumps are not adequate for performing fluid resuscitation for children in shock.

3) Further studies are needed to support comprehensive and pragmatic recommendations regarding best practice pediatric fluid resuscitation techniques.

## Abbreviations

ACCM: American College of Critical Care Medicine; HCP: Health care provider; PALS: Pediatric Advanced Life Support.

## Competing interests

Greg Harvey – No competing interests to declare, Gary Foster – No competing interests to declareAsmaa Manan – No competing interests to declare, Lehana Thabane – No competing interests to declare, Melissa Parker – Dr. Parker has received research start-up funding from McMaster and some of these funds may be used if required to cover publication costs in relation to this article. McMaster University and McMaster Children’s Hospital may benefit in reputation from publication of this article.

## Authors’ contributions

GH was primarily responsible for developing the trial protocol, including all study instruments, under the mentorship of MP. He prepared the REB submission, responded to comments, and revised all documents as required. GH assisted with the training of the research assistants and subject recruitment and was responsible for inputting all of the study data into the database. GH was involved in the data analysis and interpretation of study findings, assisted with the preparation of figures, and he drafted the first version of the manuscript. AM was responsible for trial logistical organization including the scheduling and coordination of all subject testing. AM worked under the supervision of MP and ensured that subject testing was conducted under the appropriate conditions and with scientific rigor. GF and LT assisted with trial design and statistical analysis planning. GF verified sample size requirements and provided feedback on study instruments such as the questionnaire. GF also was primarily responsible for the final data analyses. MP conceived of the research question, including the study objectives and hypotheses. She was responsible for scientific oversight of the trial including the supervision of GH and AM. MP played a significant role in development of the protocol, performed preliminary data analyses, and interpreted the study results. She revised the first draft of the manuscript prepared by GH. All authors reviewed and contributed to the submitted version of the paper.

## Authors’ information

GH is currently a third-year Pediatrics resident at McMaster University.

GF is an Assistant Professor (Part-time) of Biostatistics with the department of Clinical Epidemiology and Biostatistics. He is also a biostatistician in the Biostatistics Unit, St Joseph’s Healthcare, Hamilton.

AM is currently an MSc student in Biomedical Engineering at McMaster University.

LT is a Professor of Biostatistics, associate chair of the Department of Clinical Epidemiology and Biostatistics, associate member of the Departments of Pediatrics and Anesthesia at McMaster University (Hamilton, Ontario, Canada). He is also the Director of Biostatistics at St Joseph’s Healthcare—Hamilton, and a Senior Scientist at the Population Health Research Institute of the Hamilton Health Sciences and McMaster University.

MP is an Assistant Professor of Pediatrics at McMaster University and an Adjunct Clinical Assistant Professor of Pediatrics at the University of Toronto. She is practicing consultant in pediatric critical care and pediatric emergency medicine. Her research interest is in pediatric resuscitation, and she is supported by a Hamilton Health Sciences Research Early Career Award.

## Pre-publication history

The pre-publication history for this paper can be accessed here:

http://www.biomedcentral.com/1471-227X/13/14/prepub

## Supplementary Material

Additional file 1 **Detailed Model Description and Figures.** Text description of model and two representative figures (photos and schematic). Click here for file
