# Supplementary material for: Factors affecting pediatric isotonic fluid resuscitation efficiency: a randomized controlled trial evaluating the impact of syringe size
Source: BMC Emerg Med. 2013 Jul 24;13:14. doi: 10.1186/1471-227X-13-14 (PMC3729679; doi:10.1186/1471-227X-13-14)
Supplement: Additional file 1 — Detailed Model Description and Figures. Text description of model and two representative figures (photos and schematic). [file 1471-227X-13-14-S1.pdf]

Additional File 1: Detailed Model Description and Figures

Model Description

The model consisted of a child-sized mannequin with a 1.00 inch, 22-gauge (Insyte™ BD, Franklin Lakes, NJ), IV catheter in situ to simulate in vivo conditions. The proximal end (hub) of the IV catheter was transfixed to the hand of the mannequin in typical clinical fashion, while the distal end was connected in-line to IV conduit tubing and secured with tape. The IV tubing led in a dependent fashion to a 1 litre graduated cylinder. Connected to the hub of the IV catheter was a 7 inch long IV Catheter Extension Set (Baxter Healthcare Corporation, Deerfield, IL). The proximal end of the catheter extension set is capped with a BD Q-Syte™, which allowed for the secure attachment of Luer-Lok syringes in a “needle-less” fashion.

Figure 1: The Pediatric Fast Fluid Trial Model

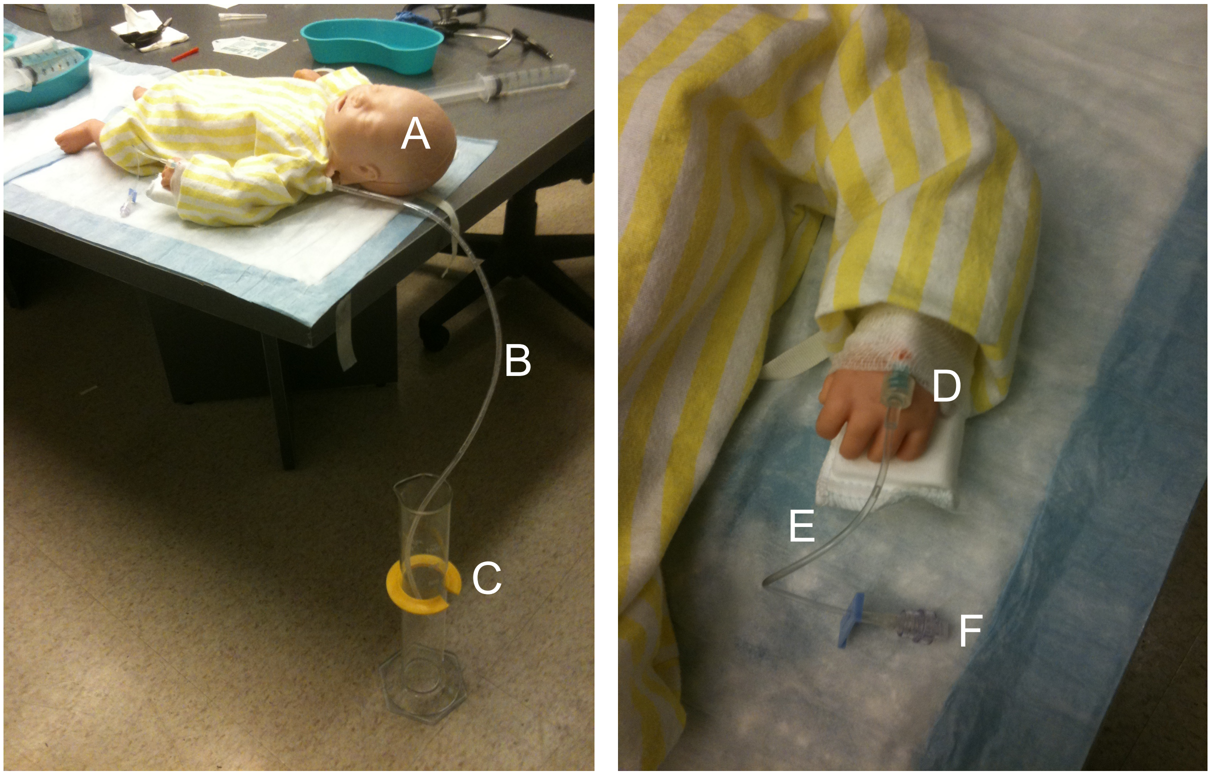

Figure Description: [Left] Model setup presented to participants in *Pediatric Fast Fluid Trial.* [Right] Close-up of IV catheter connection setup with BD-Syte attachment and extension tubing connected to 22 Gauge BD Insyte IV Catheter.

Legend:

A – Mannequin

B – Conduit Tubing

C – Graduated Cylinder

D – 22 Gauge BD Insyte IV Catheter

E – BD Extension Tubing,

F – BD Q-Syte

Figure 2: Close-up of IV Catheter Setup

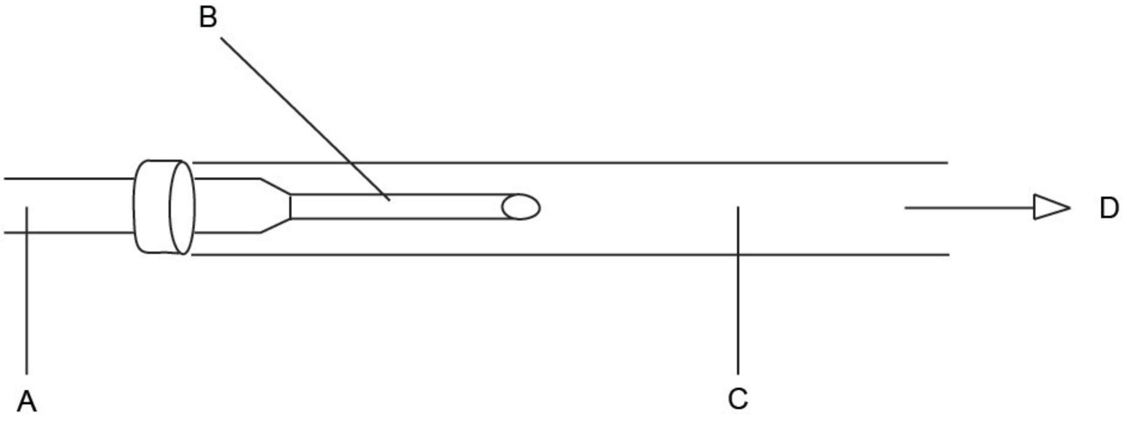

Figure Description: Representation of 22 Gauge Insyte IV catheter placement within IV conduit tubing directed towards graduated cylinder.

Legend:

A – BD Catheter Extension Tubing

B – 22 Gauge BD Insyte IV Catheter

C – Conduit Tubing

D – To Graduated Cylinder
